# Establishment and application of a preset dilution factor strategy for human chorionic gonadotropin testing in clinical laboratory

**DOI:** 10.3389/fmolb.2025.1648421

**Published:** 2025-09-03

**Authors:** Shu Chen, Qin Li, Huaqiang Liao, Mingcai Zhao, Jie Chen, Hongyu Chen, Guangjun Xiao

**Affiliations:** Department of Laboratory Medicine, Suining Central Hospital, Suining, China

**Keywords:** cost-effective optimization, preset dilution factor, automatic dilution, human chorionic gonadotropin, turn-around time

## Abstract

**Introduction:**

Automated dilution has improved the efficiency of human chorionic gonadotropin (hCG) testing, however, challenges persist regarding the cost implications of repeated testing. This study proposes the concept of cost-effective optimization, which aims to further optimize testing efficiency while maintaining controllable costs, and establishes an automated process with preset dilution factors for the hCG testing.

**Materials and methods:**

The hCG testing process at Suining Central Hospital was optimized using the Aptio™ Automation Solution (Siemens Healthineers, Erlangen, Germany) integrated with Siemens Atellica® IM1600 analyzer. A novel middleware program was developed within the DataLink V2.0 (Siemens Healthineers, Erlangen, Germany) to automate dilution factor assignment. Relevant data such as dilution factors, sample reception time and review time were captured for statistical purposes to analyze the accuracy, ability to shorten TAT, and economic benefits of hCG testing.

**Results:**

After 8 months of continuous improvement, implementation of automated hCG dilution attained 91.19% compliance rate with 19.7% reduction in in-laboratory TAT. The process achieved 75.60% compliance against the 90 min benchmark, while the preset dilution process generated 15.03% mean cost savings per test.

**Conclusion:**

In this study, a preset dilution factor program was utilized to establish an automated dilution process, achieving accurate and rapid prediction of hCG. These strategies not only improve efficiency, but also effectively reduce costs, enabling the expansion of testing items and facilitating the implementation in laboratories. Furthermore, they also help to shorten the in-hospital TAT for patients, and improve the hospital’s service quality.

## 1 Introduction

Human Chorionic Gonadotropin (hCG) is a highly glycosylated heterodimeric glycoprotein (Fournier et al., 2015) consisting of α- (93 amino acids, 14.5 kD) and β- (145 amino acids, 22.2 kD) subunits, which are noncovalently linked by charge interactions and belong to the glycoprotein hormone and cysteine-knot growth factor families (Lapthorn et al., 1994; Patel et al., 2017). hCG binds to its receptors and exerts specific roles in promoting uterine endothelial angiogenesis (Zygmunt et al., 2002), maintaining uterine muscle quiescence (Ambrus and Rao, 1994), and the metabolism of hCG by the placenta, liver, blood and kidneys determines its homeostatic levels (Nisula et al., 1989; Cole, 1997). Measurement of serum or urine hCG levels can provide important information for pregnant women and tumor patients, such as the diagnosis and monitoring of pregnancy and pregnancy-related diseases, prenatal screening, and cancer (Zhang et al., 2017; Szczerba et al., 2016). With the development of detection technology and the widespread use of immunoanalyzers, clinical hCG has gradually shifted from the previous traditional urine early pregnancy qualitative testing to the quantitative testing of serum hCG using fully automated immunoanalyzers, which makes hCG testing more rapid and accurate (Sowder et al., 2015). Chemiluminescence for testing hCG has good sensitivity and specificity, it is a commonly used method, but suffers from a narrow Analytical Measurement Range (AMR), which is less than 1,000 IU/L (Qinwei et al., 2020; Guangjun et al., 2024). Since the hCG concentration in most pregnancy samples exceeds the upper limit of the AMR, proper dilution of the samples is required prior to testing to ensure accurate results.

Clinical laboratories influence more than 70% of critical medical decisions and need to follow a strict quality management system (Inal et al., 2018; Busby et al., 2013). Turn-around Time (TAT) is one of the important indicators for evaluating the quality of clinical laboratories (Goswami et al., 2010), and the prolongation of TAT may not only delay the diagnosis and treatment of patients, but also impact patient satisfaction, in severe cases, even cause medical disputes, especially for emergency specimens. TAT is usually divided into three phases: preanalytical, in-laboratory, and postanalytical (Jandial and Gosai, 2017). In the Medical Quality Control Indicators for Clinical Laboratory Testing (National Health Commission of the People’s Republic of China, 2015), item 12 defines in-laboratory TAT as the time (in minutes) from specimen reception to report dispatch in laboratories (Howanitz et al., 1993). In-laboratory TAT reflects the work efficiency, laboratory-controllable intra- and post-testing. As laboratories become more and more intelligent, autoverification has been shown to shorten TAT, reduce labor, decrease error rates, and allow technicians to focus more on results with greater potential for error (Jones, 2013; Wang et al., 2019). However, automated dilution strategies for hCG detection in high-concentration samples require initial testing results to trigger the dilution process (Yongmei et al., 2020; Xiaolin, 2017), which essentially constitutes a repetitive testing. This method not only significantly increases the cost per sample testing, but also complicates the entire testing process and prolongs the TAT. Therefore, achieving rapid hCG quantification has become a critical issue for clinical laboratories to address, and improving the dilution process is a feasible direction. Due to cost and efficiency concerns, some laboratories remain hesitant to adopt automated dilution methods and instead rely on colloidal gold immunos-trips for manual dilution factor judgment. Although this approach optimizes the testing process to some extent, it still has significant limitations and struggles to fully meet clinical needs.

To address the optimization requirements for high-concentration hCG testing, this study proposes the concept of cost-effective optimization, which aims to solve the problem of duplicate testing faced in serum hCG testing by developing an automated program that can preset the dilution factor, so as achieve the purpose of reducing testing costs and the waiting time of patients, and to provide a reference for the quality management of clinical laboratories.

## 2 Materials and methods

### 2.1 Subjects

Using G*Power software (Version 3.1.9.7), *a priori* statistical power analysis was performed to determine the minimum sample size. For the primary hypothesis (comparing TAT differences before and after implementing the preset dilution factor strategy), the significance level was set at α = 0.05 with a power (1-β) of 0.80. Based on preliminary experiments and historical data, the pre-optimization mean TAT was estimated at 100 min (SD = 20 min), and the post-optimization mean TAT at 90 min, yielding an effect size d = 0.50. The analysis indicated a minimum requirement of 64 samples per group (total N = 128) to achieve adequate statistical power.

Firstly, 269 pregnancy specimens in the laboratory of Suining Central Hospital were collected to analyze the relationship between gestational weeks and dilution factors. Subsequently, a total of 13,636 testing results of hCG testing in the laboratory between 1 January 2024 and 1 September 2024 were retrieved from the backend of Siemens Atellica® IM1600 analyzer (Siemens Healthineers, Erlangen, Germany). 458 specimens were screened as experimental and control groups respectively, and the selection was random. Relevant data such as the dilution factors, sample reception time, and review time were collected. Specimens with abnormal TAT and gestational weeks other than 5–40 weeks were removed, leaving 454 specimens in the experimental group and 395 in the control group.

### 2.2 Methods

Based on the relationships obtained statistically from the hospital’s previous data, the dilution factor judgment logic rules were established. In order to apply logic rules to the clinical laboratory, the hCG preset dilution factor program was developed and embedded into the DataLink V2.0 (Siemens Healthineers, Erlangen, Germany). This integration facilitated connectivity between the Siemens Atellica® IM1600 analyzer and the Aptio™ Automation Solution (Siemens Healthineers, Erlangen, Germany), establishing an automated hCG testing process with intelligent dilution factor assignment.

The experimental group used the strategy of preset dilution factor, while the control group used immunos-trips for judgment (representing the laboratory’s default workflow prior to this optimization study). Samples from gestational weeks 5–40 were processed using the preset dilution factor process. Given that hCG concentrations exhibit significant variation and rapid changes during early gestation (<5 weeks), establishing reliable correlations between gestational week and dilution factor proves challenging. Consequently, samples with gestational weeks <5 underwent manual dilution via colloidal colloidal gold immunos-trips.

Compare the two groups and analyze the ability of shortening TAT, the accuracy of testing, and economic benefits. For the economic benefit analysis, assuming that the price of the anti-hCG testing reagent is α yuan/testing, the Atellica IM ThCG kit (chemiluminescence immunoassay, CLIA) can be regarded as 31.91 α yuan/testing based on the purchase price. Therefore, the difference in additional cost between experimental and control groups can be calculated.

### 2.3 Statistical analysis

Python 3.9 (64-bit) was used for the computer program, while SPSS 26 and Origin 2018 (64-bit) platforms executed statistical analyses. Continuous variable (TAT) distribution normality was evaluated using Kolmogorov-Smirnov test. Gestational week, median TAT, and mean TAT comparisons between cohorts employed independent t-tests, with significance threshold set at α = 0.05 (two-sided test; P < 0.05 deemed statistically significant).

The research related to human use has complied with all the relevant national regulations, institutional policies, and in accordance with the tenets of the Helsinki Declaration, and has been approved by the authors’ Institutional Review Board or equivalent committee (Medical Research Ethics Committee of Suining Central Hospital, KYLLMC20240018). Informed consent was obtained from all individuals included in this study, or their legal guardians or wards.

## 3 Results

### 3.1 Establishment of the preset dilution factor process

In the clinical laboratory of Suining Central Hospital, the hCG testing process begins with barcode labeling of collected specimens, delivered to the laboratory and received via the rail system. Through the Labbot sorting system (Labbot, Jiangsu, China), they are automatically sorted and pneumatically transported to the AptioTM fully automated chemical immunization line for checking. Next, specimens are transported to the Siemens Atellica® IM1600 analyzer for testing. Results are verified and interpreted, and subsequently, reports are dispatched to doctors and patients. We analyzed previous data from this hospital to analyze the association between gestational weeks and dilution factors. The hCG dilution factors at 2–4 weeks varied widely, including undiluted, 10-fold and 100-fold, making it difficult to determine the sample dilution factor for early pregnancy subjects by the gestational week; most of the hCG samples at 5–6 weeks were diluted to 100-fold; all of the samples at 7–12 weeks were diluted to 200-fold; and the dilution factors at 13–40 weeks declined again to 100-fold, as shown in Table 1. Logic rules for dilution factor judgment were developed based on the above statistics.

**TABLE 1:** Relationship between gestational weeks and hCG dilution factors.

Gestational weeks	Dilution factors	Sample size percentage
2–4	10	27.27%
5–6	100	77.42%
7–8	200	100%
9–10	200	100%
11–12	200	100%
13–27	100	100%
28–40	100	100%

**FIGURE 1:**
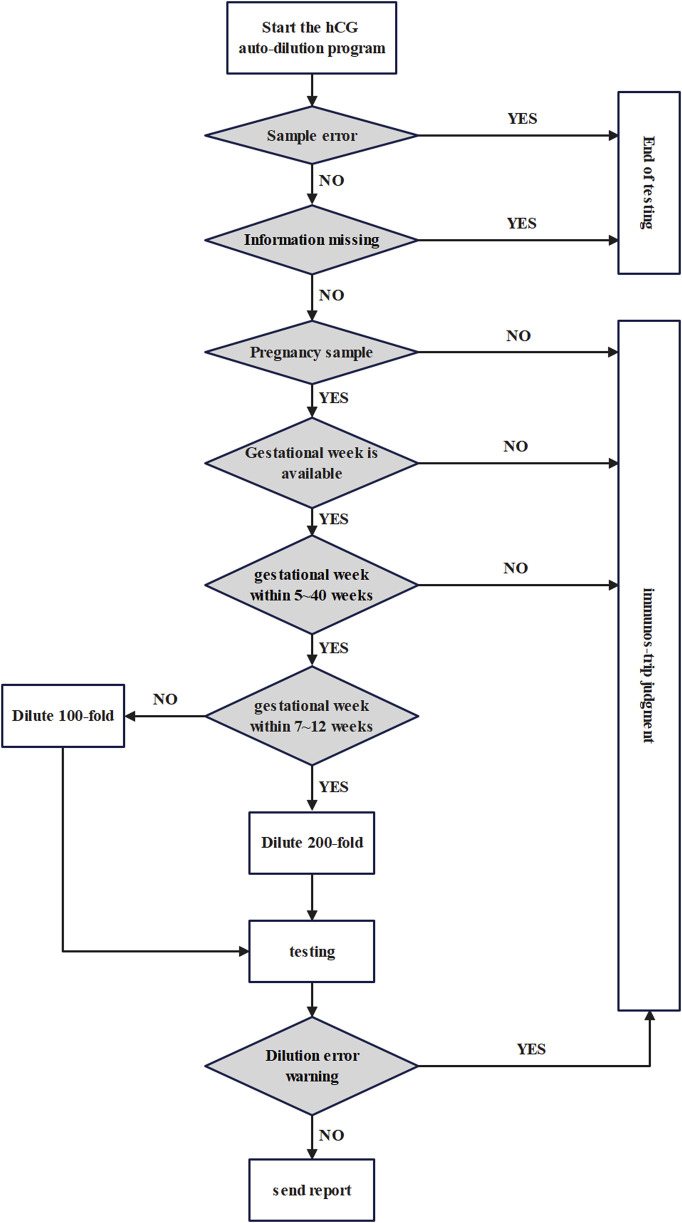
Flowchart of preset dilution factor process for hCG.

The flowchart of the preset dilution factor process of hCG is shown in Figure 1. Firstly, samples with sample errors, missing information, non-pregnant patients, unavailable gestational week information, and samples with gestational weeks other than 5–40 gestational weeks are pushed out directly through the I/O module for manual dilution judgement using the colloidal gold immunos-trip. Eligible samples continue to be automatically diluted, with the dilution factor determined by the logical rule of 100-fold dilution for samples at 5-6 and 13–40 gestational weeks, and 200-fold dilution for samples at 7–12 gestational weeks. If the dilution factor is misjudged, the sample will be pushed out for manual judgment. Finally, a testing report with correct results will be issued.

### 3.2 Analysis of gestational week data

Gestational week data were analyzed for the experimental and control groups as shown in Table 2. The experimental results showed that there was no significant difference between gestational week data of the two groups (*P* = 0.86, >0.05), which could be further analyzed by TAT.

**TABLE 2:** Comparison of gestational week data.

Parameter		Experimental group	Control group
Sample n		454	395
Mean value		6.95	6.76
SD		3.10	3.26
t		0.87	
*P*-Value		0.86	
95% confidence interval	Lower limit	−0.24	
	Upper limit	0.62	

### 3.3 Analysis of in-laboratory TAT

**FIGURE 2:**
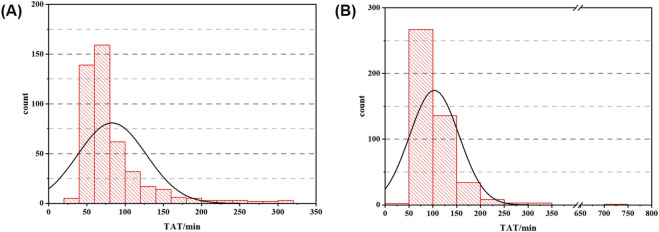
TAT distribution data graph (**(A)** histogram of experimental group; **(B)** histogram of control group).

As shown in Table 3, the in-laboratory TAT median for hCG was shortened from 89.62 min to 68.27 min after the improvement. The TATs at 5%, 25%, 75%, and 95% percentiles were 45.95 min, 57.33 min, 88.72 min, and 178.75 min in the experimental group; 60.11 min, 72.55 min, 119.44 min, and 182.23 min in the control group, respectively. In addition, the mean value was 82.92 min for the experimental group and 103.90 min for the control group. Combined with the t-test results, which were statistically significant at the *P* = 0.05 level (*P* < 0.001, F = 2.088, t = −6.127), there was a significant difference between the means of the experimental group and control group. The distribution and normality testing of TAT data are shown in Figure 2.

**TABLE 3:** Descriptive analysis of in-laboratory TAT for hCG (min).

Group	Mean value	Median	Range	5%	25%	75%	95%
Experimental group	82.92	68.27	33.48–313.07	45.95	57.33	88.72	178.75
Control group	103.90	89.62	25.93–749.77	60.11	72.55	119.44	182.23

**FIGURE 3:**
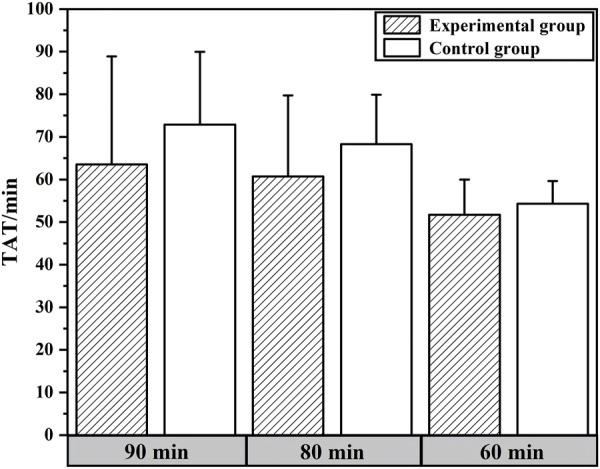
Comparison of TAT values between experimental and control groups under different expectation levels.

The expectation analysis data for in-laboratory TAT are shown in Table 4. When an expected value of 90 min was set for the in-laboratory TAT for hCG, 75.60% of samples in the experimental group and only 51.02% in the control group met the expectation. When TATs of the experimental and control groups were each compared to 90 min using the t-test, significant differences were found at the *P* = 0.05 level. Specifically, the in-laboratory TAT of the experimental group was significantly lower than 90 min, while the control group was significantly higher than 90 min. Furthermore, when both groups were separately evaluated against thresholds of 90 min, 80 min, and 60 min, t-test results indicated statistically significant differences between the experimental and control groups at both 90 min and 80 min thresholds. The comparison of TAT values between the experimental and control groups at 90 min , 80 min , and 60 min is shown in Figure 3.

**TABLE 4:** In-laboratory TAT expectation analysis for hCG (90, 80, and 60 min were taken as the expected values, respectively).

Time	Experimental group		Control group		P	95% [CI]	
	Sample n	Compliance rate	Sample n	Compliance rate		Lower limit	Upper limit
90 min	344	75.60%	200	51.02%	<0.001	−11.37	−7.30
80 min	303	66.59%	146	37.24%	<0.001	−9.46	−5.67
60 min	144	31.65%	18	4.59%	0.078	−5.61	0.30

### 3.4 Analysis of compliance rate and economic benefits

As shown in Table 5, among the 454 samples in the experimental group, 414 were correctly assigned dilution factors, indicating an hCG compliance rate of 91.19% under the automated preset dilution factor strategy, and the rework rate of 8.81%. Compared to the 7.00% rework rate observed under the immunos-trip strategy, the experimental group achieved a 15.03% reduction in additional costs.

**TABLE 5:** data of compliance rate and economic benefits (α is the price of the anti-hCG testing reagent).

	Compliance rate	Rework rate	Additional cost	Cost rate
Experimental group	91.19%	8.81%	31.91α×8.81%	84.97%
Control group	93.00%	7.00%	31.91α×7%+α	100%

## 4 Discussion

The main mission of the clinical laboratory is to ensure the quality of clinical diagnosis by providing timely and accurate results (Getawa et al., 2022). Although most workflows in laboratories are well designed, there are still inefficiencies that affect quality, such as unnecessary duplication of services, long waiting times and delays. Nowadays, hCG testing faces two major challenges: firstly, the sensitivity limitation of the instrument, specifically its narrow AMR, which restricts the ability to test high-concentration hCG samples, and the “hook effect” that arises when the hCG concentration is close to or even exceeds the peak value (Griffey et al., 2013; Benamour et al., 2024; Cormano et al., 2015). As the hCG concentration increases, the color of the testing line becomes weaker, presenting a false-negative result due to high antigen concentration (Baudin and Pilon, 2019), which needs to be corrected by diluting the sample (Berger and Sturgeon, 2014); secondly, there is the issue of sample dilution. The automatic dilution strategy, which selects a dilution factor based on the initial testing result (i.e., the “test-dilute-retest” mode), leads to additional costs and prolongs the TAT. Therefore, some hospitals have optimized the dilution process. The use of colloidal gold immunos-trip for dilution factor determination prior to sample testing is an effective way to reduce the cost of duplicate testing (Guangjun et al., 2019; Nwabuobi et al., 2017). However, the method increases the risk of staff exposure to the specimen, and due to the varying skills of operators, it is easy to make errors in judgment by observing the discoloration reaction of immunos-trip with the naked eye. In addition, the use of immunos-trip judgment also has the problem of cumbersome workflow. Since the TAT for hCG testing in Suining Central Hospital failed to meet the required 90 min standards, particularly for emergency specimens, this study performed a secondary optimization based on immunos-trip judgment and developed an automated program with preset dilution factors, which assigns the dilution factor automatically based on gestational week information prior to sample testing. This method not only eliminates the additional costs associated with repeated testing but also further reduces the cost of immunos-trip usage and shortens the TAT. Additionally, it effectively minimizes errors introduced by manual operations.

In early pregnancy, hCG is produced primarily by differentiated syncytiotrophoblasts and is a key embryonic signal required to maintain pregnancy (Zygmunt et al., 2002; Ambrus and Rao, 1994). The hCG level reaches 25 mIU/mL after 10 days of pregnancy and increases exponentially thereafter. During the first 4 weeks of pregnancy, hCG promotes the secretion of progesterone and estradiol by converting the postovulatory ovary into the gestational corpus luteum, and hCG concentrations double approximately every 2–3 days. The rate then slows down and reaches a peak of 30,000-200,000 mIU/mL in 8–10 weeks before declining to a much lower steady-state level (Lee et al., 2013; Pocius et al., 2017; Pocius et al., 2015; Fan et al., 2017). The hCG level below 5 mIU/mL excludes pregnancy, an increase in hCG that is lower than expected indicates an abnormal pregnancy, such as miscarriage and ectopic pregnancy, while a decrease usually indicates pregnancy failure (Regan and Rai, 2000). This shows that the gestational week is closely related to the hCG level and that regular monitoring of hCG is crucial for pregnant women. This study established a relationship model between gestational week and hCG concentration, achieving a compliance rate of 91.19%.

Under the preset dilution factor process, an average of 20.98 min was saved per specimen due to the reduction of sample push-out/push-in and manual judgment steps using immunos-trips. The hospital receives approximately 1,250 hCG specimens per month, which represents 437.08 h per month that will be saved. There was a significant difference (*P* < 0.05) in TAT between the experimental and control groups under t-test. Additionally, the improved process better meets the 90 min expectation. Meanwhile, these strategies also reduce laboratory costs due to further savings on immunos-trips consumption, saving an average of 15.03% additional cost per testing, which brings significant economic benefits and provides a more efficient solution for clinical laboratories. Schachinger et al. (2024) established an automated pre-dilution setup for Von Willebrand Factor (VWF) activity assays. Although this system replaced manual dilution in VWF testing, it still requires an initial test to trigger the dilution process, essentially maintaining the “test-dilute-retest” mode. The breakthrough of our study lies in its capacity to not only automate dilution but also accurately predict dilution factors prior to dilution (91.19% compliance rate). This strategy eliminates redundant testing from the source and significantly enhances efficiency compared to conventional automated dilution systems. Ialongo et al. (2017) implemented an artificial neural network for smart management of sample dilution, predicting the initial dilution status for serum free light chain (sFLC) testing. Compared to the initial stepwise dilution strategy used by automated analyzers, their approach reduced wasted testing for serum κ-FLC and λ-FLC by 69.4% and 70.8%, respectively. Our preset dilution factor strategy demonstrates optimization results comparable to this artificial neural network strategy: reducing the average TAT by 19.7% and lowering costs by 15.03%, thereby streamlining the testing workflow and conserving resources.

Finally, we would like to emphasize some strengths of this study and outline future research directions. This study creatively established an accurate correlation between gestational week and hCG concentration, and a program was developed that enables selection of the dilution factor. These strategies not only shortened the TAT of hCG specimens by 20.98 min, but also reduced the additional cost by 15.03%. Compared with the repeated testing of automatic dilution and the immunos-trip judgment, they have significant advantages in shortening the TAT and saving labor and material costs, which improve the responsiveness and quality of healthcare services, and bring new thinking to clinical testing. Future research should explore the potential applicability of this preset dilution factor strategy beyond obstetric testing—such as for tumor markers including Tg, AFP, CEA, and PSA. The core technical challenge lies in establishing robust correlations between predictive variables (e.g., disease stage or tumor type) and analyte concentrations to improve compliance rates. Moreover, AI-enhanced big data analytics and the development of regional integrated centers represent critical research directions. Subsequent studies should leverage machine learning to model logical relationships between predictive variables and analyte concentrations (Huang et al., 2022; Barnett-Itzhaki et al., 2020), enabling precise dilution factor assignment. Concurrently, establishing regional integrated centers would standardize the implementation of preset dilution process.

## 5 Conclusion

Overall, the preset dilution factor strategy addressed the process optimization of hCG testing in clinical laboratories, which significantly shortened the TAT and reduced testing costs. This not only improves laboratory efficiency and testing accuracy, making it valuable for clinical laboratory automation and intelligence, but it also provides patients with faster and more accurate personalized diagnosis and treatment services, thereby helping to shorten the waiting time of patients and improve the hospital visit rate. This improvement has had a positive impact, both from the perspective of patients and hospital operations, enhancing the overall quality of hospital services.

## Data Availability

The original contributions presented in the study are included in the article/Supplementary Material, further inquiries can be directed to the corresponding author.
